# Oncogenic fusion of BCAR4 activates EGFR signaling and is sensitive to dual inhibition of EGFR/HER2

**DOI:** 10.3389/fmolb.2022.952651

**Published:** 2022-08-23

**Authors:** Kieun Bae, Jin Hee Kim, Ja Young Lee, Sun-Young Kong, Yun-Hee Kim, Sunshin Kim, Kyong-Ah Yoon

**Affiliations:** ^1^ College of Veterinary Medicine, Konkuk University, Seoul, South Korea; ^2^ College of Health Science, Cheongju University, Cheongju, South Korea; ^3^ Research Institute, National Cancer Center, Goyang, South Korea; ^4^ National Cancer Center Graduate School of Cancer Science and Policy, Goyang, South Korea

**Keywords:** oncogenic fusion, CD63-BCAR4, EGFR activation, lapatinib, canertinib

## Abstract

We previously reported CD63-BCAR4 fusion as a novel oncogene that significantly enhanced cell migration and metastasis in lung cancer. To identify effective inhibitors of metastatic activity induced by BCAR4 fusion, we screened a drug library of 381 FDA-approved compounds. The effect of drugs on cell migration was evaluated by monitoring wound healing. Drugs that decreased the cellular mobility of fusion-overexpressing cells compared with that of control cells were selected as candidates. Library screening revealed that erlotinib, canertinib, and lapatinib demonstrated inhibitory effects on cell migration. Activation of the EGFR signaling pathway was detected after ectopic expression of CD63-BCAR4 in normal bronchial epithelial cells, as observed by the increased phosphorylation of tyrosine residues in the EGFR protein. We also confirmed increased levels of the phosphorylated EGFR protein in resected tumors from mice injected with CD63-BCAR4 overexpressing cells. Tyrosine kinase inhibitors (TKIs) of the EGFR family significantly inhibit the migration of BCAR4 fusion-overexpressing cells and induce apoptosis at high concentrations. Among the EGFR family TKIs, canertinib, a dual EGFR/HER2 inhibitor, showed the best inhibitory effect on the migration and viability of BCAR4 fusion-overexpressing cells. We examined the effect of canertinib *in vivo* using a mouse xenograft model. Oral administration of canertinib to xenografted mice reduced tumor growth induced by the CD63-BCAR4 fusion gene. In addition, canertinib treatment restored E-cadherin expression and reduced the expression of epithelial–mesenchymal transition regulatory factors such as Slug and Snail. Taken together, these results suggest that EGFR/HER2 inhibitors are potential therapeutic options for BCAR4 fusion-harboring lung cancer patients, even in the absence of EGFR mutations.

## 1 Introduction

The presence of actionable mutations has led to clinical benefits for a subset of patients who can be effectively treated with targeted therapies. Patients with lung cancer have greatly benefited from target identification and the development of therapeutics (Cancer Genome Atlas Research, 2014; Jordan et al., 2017). Oncogenic fusions have been identified as crucial alterations that lead to cancer and are therapeutic targets. Since the fusion of echinoderm microtubule-associated protein like-4 (EML4)−anaplastic lymphoma kinase (ALK) was discovered in non-small cell lung cancer (NSCLC), ALK rearrangements have been reported in 3–8% of lung adenocarcinomas (Soda et al., 2007; Mano, 2008; Devarakonda et al., 2015). The *rearranged during transfection* proto-oncogene (RET) and *v-ros* proto-oncogene (ROS1) have also been identified as fusion types that contribute to lung cancer by leading to constitutive activation of kinase (Davies and Doebele, 2013; Tsuta et al., 2014). Lung cancer patients harboring therapeutically actionable fusion genes, such as *ALK, RET,* and *ROS1*, have experienced clinical benefits owing to the development of effective targeted therapies with crizotinib being foremost (Chao et al., 2012; Shaw et al., 2014; Solomon et al., 2014). The discovery of new targets and identification of effective inhibitors against them are important in the field of cancer research. We previously reported *CD63-BCAR4* fusion as a novel oncogene that significantly enhanced cell migration and metastasis in lung cancer (Bae et al., 2021). The *CD63-BCAR4* fusion gene discovered in Korean patients with lung adenocarcinoma consists of exons 1-3 of *CD63* and exon 4 of *BCAR4*. Ectopic expression of the CD63-BCAR4 protein in normal bronchial epithelial cells resulted in enhanced cell proliferation and migration compared to that in control cells. Tumorigenicity and metastasis induced by the CD63-BCAR4 fusion protein have been demonstrated in a mouse xenograft model.

*CD63-BCAR4* fusion gene was also discovered in a Chinese female patient with lung adenocarcinoma (Wang et al., 2018). Other fusion transcripts with *BCAR4* have been reported in several studies. *ERBB3-BCAR4* fusion has been reported in two patients: one patient from the lung adenocarcinoma project of the Cancer Genome Atlas consortium (TCGA-LUAD) and another from a whole-genome sequencing study of Chinese lung adenocarcinoma (Cancer Genome Atlas Research, 2014; Wu et al., 2015). Recently, *LITAF-BCAR4* fusion was detected in a patient with infiltrating lobular breast cancer that resulted in BCAR4 overexpression (Vasmatzis et al., 2020). In patients with lung cancer harboring fusion transcripts of *BCAR4*, no other known activating mutations of *EGFR* and *KRAS* genes were found. Owing to the absence of *EGFR* mutations, these patients were not considered candidates for treatment with EGFR TKIs.

*BCAR4* gene is a long noncoding RNA (lncRNA) that also encodes a short protein; therefore, it has both coding and noncoding functions. BCAR4 was originally identified from the genes responsible for tamoxifen resistance in breast cancer cells as named as a breast cancer anti-estrogen resistance 4 gene (Meijer et al., 2006). BCAR4 overexpression, which was associated with tamoxifen resistance and poor outcomes, sensitized breast cancer cells to lapatinib (Godinho et al., 2012). Overexpressed BCAR4 has been reported in cervical cancer, non-small cell lung cancer (NSCLC), and gastric cancer, suggesting that it may be a biomarker for poor prognosis (Wang et al., 2017; Yang et al., 2018; Zou et al., 2018). BCAR4 expression promoted the proliferation and motility of cervical cancer cells by enhancing epithelial–mesenchymal transition (EMT) (Zou et al., 2018). BCAR4 also supported invasion and metastasis of NSCLC, resulting in poor prognosis (Gong et al., 2017; Li et al., 2017; Yang et al., 2018). BCAR4 has also been reported to activate Wnt/β-catenin signaling in enhancing cell proliferation (Ouyang et al., 2017).

Exogenous CD63-BCAR4 fusion protein induced tumorigenicity in a mouse xenograft model and promoted metastatic tumors in the livers and lungs of xenografted mice in our previous study. In the present study, we have sought identify an effective inhibitor of the oncogenic function of the BCAR4 fusion protein. We aimed to screen a drug library of FDA-approved compounds and subsequently investigate the therapeutic potential of these inhibitors through *in vitro* and *in vivo* analyses. Our results demonstrate the inhibitory effect of small-molecule inhibitors of EGFR and HER2 in BCAR4 fusion-overexpressing cells. Thus, BCAR4 fusion might be a potential therapeutic target for EGFR/HER2 inhibition.

## 2 Materials and methods

### 2.1 Reagents

A drug library containing 381 FDA-approved drugs was purchased from Selleckchem (Houston, TX, United States). Compounds were dissolved in DMSO and stored at −20°C. Drugs diluted with RPMI 1640 medium were used to treat cells at a final concentration of 5 μM. The selected drugs, erlotinib, canertinib and lapatinib, were purchased from Selleckchem. EGFR-TKIs were used in the experiments at a concentration of 0.5–20 μM. The primary antibodies for EGFR, phospho-EGFR(Y1068), HER2, phospho-HER2(Y1248), vimentin, Snail, Slug, cleaved-PARP, and HA-tag were purchased from Cell Signaling Technology (Danvers, MA, United States). E-cadherin, N-cadherin, and active-caspase-3 were purchased from Santa Cruz Biotechnology (Dallas, TX, United States). β-actin was purchased from Sigma-Aldrich (St. Louis, MO, United States).

### 2.2 Stable cell lines

We established stable cell lines that overexpressed *CD63-BCAR4* fusion, *BCAR4* (HE601934.1) and an empty vector from BEAS-2B, an immortalized human bronchial epithelial cell line. The coding regions of *CD63-BCAR4* fusion and *BCAR4* were overexpressed using a lentiviral vector with a hemagglutinin (HA) tag, CMV promoter, and green fluorescent protein as a transduction marker. Authentication of the cell lines was assessed using short tandem repeat analysis, and mycoplasma infection was tested using PCR. Each cell line was cultured at 37°C in 5% CO_2_ in RPMI 1640 with 10% fetal bovine serum and 1% penicillin-streptomycin (Thermo Fisher Scientific, Waltham, MA, United States).

### 2.3 Migration assay

Stable cell lines were seeded in 96-well Essen ImageLock plates at a density of 20,000 cells/well. Then, the monolayer cells were scratched using a 96-pin wound maker. Subsequently, the wound-scratched cells were treated with drugs for 48 h. Cell migration was monitored using the IncuCyte™ Live-Cell Imaging System (IncuCyte live-cell Essen BioScience Inc., Ann Arbor, MI, United States). Wound images were acquired by automatically scanning the plates every 2 hours, and the collected data were analyzed using the IncuCyte software system.

### 2.4 Western blot analysis, Immunohistochemistry, and Immunocytochemistry

For western blot analysis, cells were lysed in RIPA buffer (Cell Signaling Technology) containing protease and phosphatase inhibitor cocktails. The protein concentrations were determined using a protein assay reagent (Bio-Rad Laboratories, Hercules, CA, United States). Proteins were separated on sodium dodecyl sulfate-polyacrylamide gels using electrophoresis. The separated protein was transferred to PVDF membranes (Merck Millipore, Carrigtwohill, Ireland), and the blots were blocked for 1 h with 5% skim milk (BD Biosciences, Franklin Lakes, NJ, United States) in Tris-buffered saline containing 0.1% tween-20. Thereafter, the blots were incubated overnight with primary antibodies. Proteins were visualized using a horseradish peroxidase-conjugated secondary antibody (Cell signaling Technology) and an enhanced chemiluminescence (ECL) reagent (Bio-Rad Labaratories). β-Actin was used as a loading control.

For immunocytochemistry, cultured cells were seeded at a density of 20,000 cells on Lab-Tek ?R?R glass chamber slides (Thermo Fisher Scientific, Rochester, NY, United States). Subsequently, the cells were fixed in a 4% formaldehyde solution for 10 min at room temperature and permeabilized by treatment with PBS containing 0.1% Triton X-100 for 5 min. The cells were blocked with 1% bovine serum albumin (BSA) for 1 h and incubated with primary antibodies overnight. The cells were stained with a goat anti-mouse and or anti-rabbit IgG conjugated to an Alexa Fluor Plus 647 secondary antibody (Invitrogen, Waltham, MA, United States) and viewed using a confocal laser scanning microscope (LSM 900 with Airyscan2; Carl Zeiss AG, Oberkochen, Germany). Images were captured using an image analysis software (ZEN; Carl Zeiss, Munich, Germany).

### 2.5 Hematoxylin and eosin staining and immunohistochemistry

To investigate the xenografted mouse tissues, the tissues were embedded in paraffin and cut into 4 μm thick sections for histology and immunohistochemistry. Tissue sections were stained with hematoxylin and eosin (H&E). The images were scanned on a brightfield whole slide using a Slide Scanner Vectra Polaris ^TM^ (PerkinElmer, Waltham, MA, United States).

We also stained the tumor and liver tissue sections with specific antibodies after blocking with 1% BSA. The sections were then incubated with a biotinylated secondary antibody. Staining was performed with 3,3′-diaminobenzidine (DAB) substrate and the tissue sections were counterstained with hematoxylin. Positive DAB signals in the IHC images of phosphorylated EGFR and HER2 were quantified by using ImageJ software (NIH, Bethesda, MD, United States). ImageJ software analyzed the staining intensity of at least five fields of view and calculated the percentage of total area occupied by positive cells.

### 2.6 Cytotoxicity and apoptosis assay

Cytotoxicity was monitored using the LDH-Glo™ cytotoxicity assay (Promega, Madison, WI, United States). Briefly, cells were plated in 96-well plates at a density of 5,000 cells and treated with EGFR-TKIs for 24 h. Supernatants were incubated with the reagent for 60 min in the dark. After incubation, luminescence for cell cytotoxicity was measured using a GloMax NAVIGATOR (Promega). All experiments were performed in triplicate.

Induction of apoptosis by EGFR-TKIs was assessed using the APC-Annexin V assay according to the manufacturer’s instructions (BD Biosciences, United States). Additionally, 7-Amino-Actinomycin D (7-AAD) (BD Biosciences) was added to the binding buffer to stain the necrotic and dead cells. The resuspended cells in the stain solution were gently vortexed and incubated for 15 min at room temperature in the dark. Stained cells were detected using a flow cytometer (BD FACSCalibur™, Becton Dickinson, United Kingdom) and analyzed using the FlowJo v10.7.2 system.

### 2.7 RNA interference (siRNAs) and cell viability assay

Predesigned small-interfering RNAs (siRNAs) of EGFR, HER2, BCAR4, and a non-target negative control (Silencer^®^, AM4635) were purchased from Invitrogen. Transfection was performed using the Lipofectamine 2000 (Invitrogen) according to the manufacturer’s instructions. CD63-BCAR4 overexpressing cells were seeded in a 6-well plate (2 × 10^5^ cells per well) and treated with siRNAs 0.05 nmol for 48 h. The cells were then harvested for further experiments.

Transfected cells were seeded in 96-well plates and incubated at 37°C in a humidified atmosphere with 5% CO_2_. After 48 h, cell viability was assessed using the CellTiter-Glo® luminescent cell viability assay (Promega).

### 2.8 *In Vivo* tumorigenicity

NOD.Cg-Prkdcscid II2rgtm1Sug/Jic (NOG) mice were purchased from the Central Institute for Experimental Animals (CIEA, Tokyo, Japan), and 4–5 mice were housed per cage in an animal facility under controlled temperature (22–24°C) and humidity (40–60%) conditions. CD63-BCAR4 overexpressing BEAS-2B cells (5 × 10^6^) were injected subcutaneously into 6-week-old NOG mice (average body weight 18 g ± 1.5 g) after inducing anesthesia by 1.5–3% isoflurane inhalation (HanaPharm, Seoul, Korea). Tumors and body weights were measured weekly, and tumor volumes were calculated (length × width^2^ × 0.5) (Tomayko and Reynolds, 1989). When the tumor size reached 150 mm^3^, canertinib was administered *via* oral gavage at a concentration of 5 mg/kg in 30% propylene glycol, 5% Tween 80, and 65% D5W Monday through Friday for 6 weeks. Mice were sacrificed using carbon dioxide gas before the tumors reached 1,500 mm^3^ in volume. All animal experiments were performed with the approval of the Institutional Animal Care and Use Committee (IACUC) of our institute (NCC-18-318C and KU19010).

### 2.9 Statistical analysis

Statistical significance was tested using unpaired *t*-test or ANOVA test for comparisons between two or more groups. All reported *p*-values were two-sided, and graphs were generated using the GraphPad Prism software (GraphPad Software Inc., San Diego, CA, United States). Significant differences are marked by *(*p* < 0.05), **(*p* < 0.005), and ***(*p* < 0.0005).

## 3 Results

### 3.1 FDA-approved drug library screening identifies EGFR inhibitors for the migration induced by CD63-BCAR4 fusion

**FIGURE 1:**
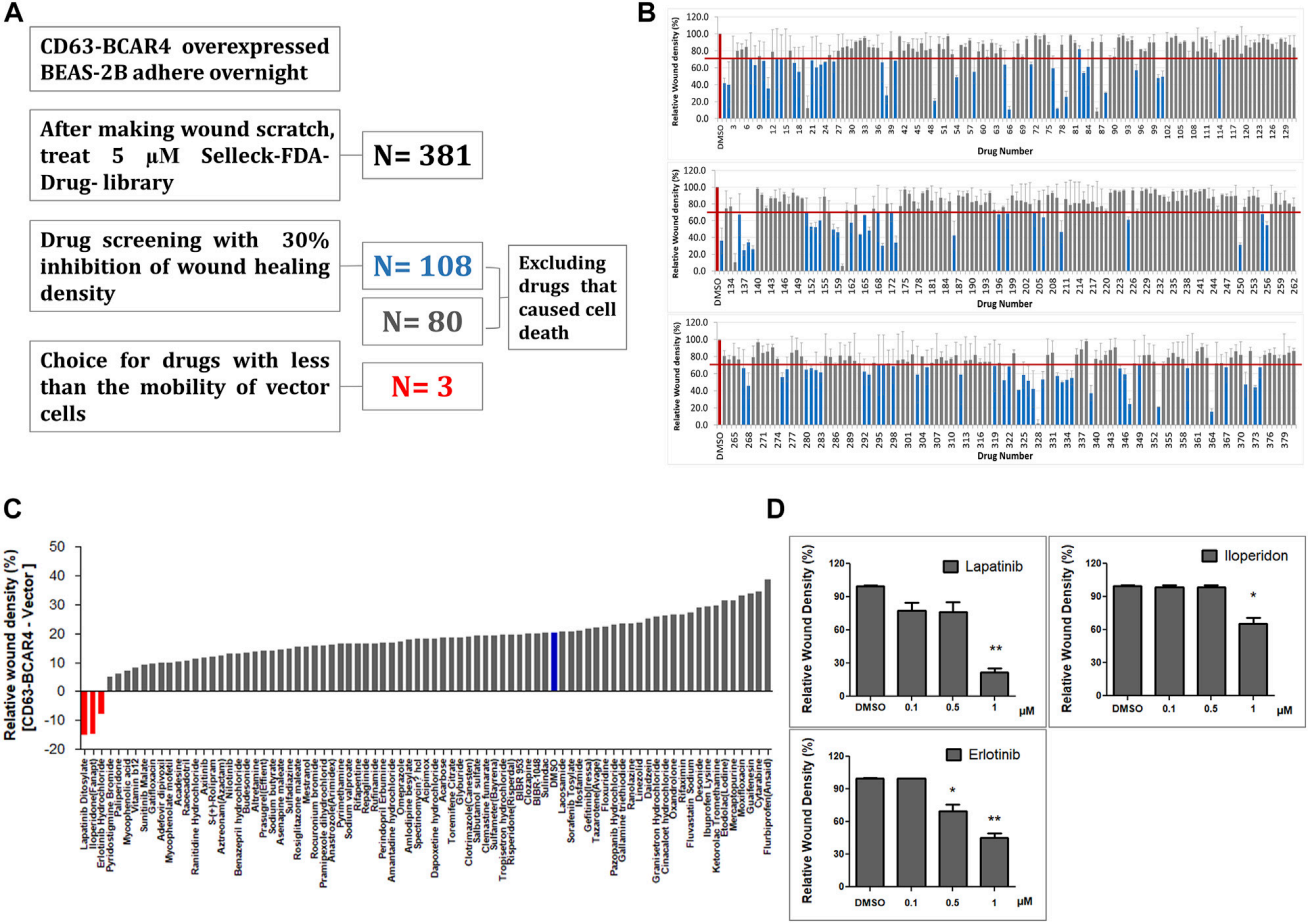
Drug library screening to identify inhibitors of cell migration induced by CD63-BCAR4 fusion. **(A)** Procedure to screen the drugs that effectively inhibit wound healing of BCAR4 fusion-overexpressing cells. **(B)** The bar graph represents the primary screening results of 381 FDA-approved drugs to compare inhibitory effect on cell migration. **(C)** Drug effects were simultaneously evaluated in both control cells (empty vector overexpressing cells) and CD63-BCAR4 overexpressing cells. The bar graph shows the ranking of candidate drugs that better inhibited the migration of BCAR4 fusion-overexpressing cells compared with the control cells. **(D)** Inhibitory effects of three drug candidates on wound healing were compared in a dose-dependent manner. Replicated experiments were performed and statistical significance are marked with * (*p* < 0.05), and ** (*p* < 0.005).

The effects of 381 FDA-approved drugs on cell migration were evaluated by monitoring cell mobility to heal the wound site. First, 108 drugs that inhibited cell migration by less than 30% compared with the control were selected (Figures 1A,B). Twenty-eight drugs that induced excessive cell death, making it difficult to compare their effects on cell migration, were excluded from the subsequent step. Next, only drugs that showed inhibitory effects on fusion-overexpressing cells, but not on control cells, were selected as candidates. The top three drugs were lapatinib, iloperidone, and erlotinib (Figure 1C). The concentration-dependent effects of the three drugs were analyzed, and lapatinib and erlotinib showed relatively good inhibitory effects (Figure 1D).

### 3.2 CD63-BCAR4 fusion activates EGFR and downregulates HER2

**FIGURE 2:**
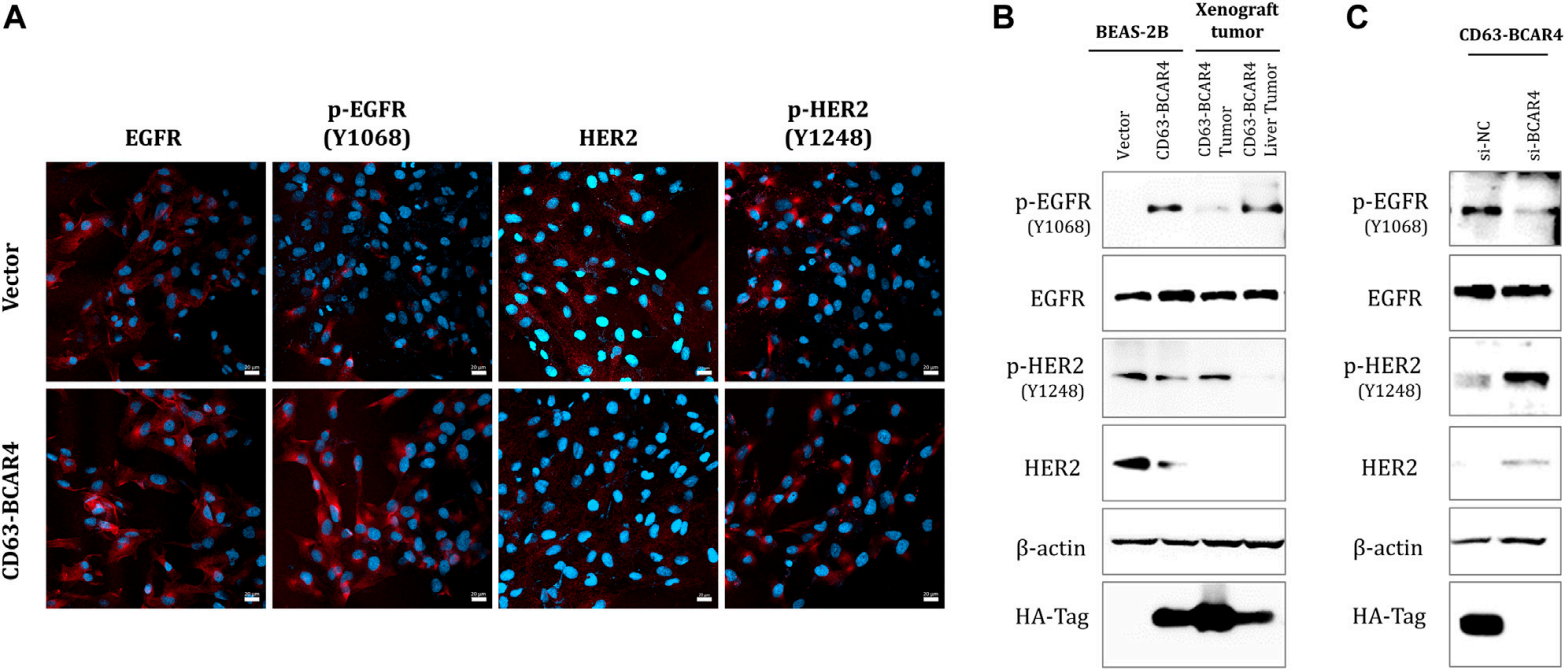
Expression of EGFR and HER2 proteins in CD63-BCAR4 overexpressed cells. **(A)** EGFR, p-EGFR(Y1068), HER2, p-HER2 (Y1248) (red) and Hoechst 33,342 (blue) nuclear staining in CD63-BCAR4 overexpressing cells were determined using immunocytochemistry. **(B)** Western blot showing protein levels in overexpressed cells, tumors, and metastatic liver tumors from xenograft mice. **(C)** Protein expression level in cells was evaluated by transfection of siRNA targeting BCAR4 (si-BCAR4) and a negative control (si-NC).

To evaluate the inhibitory effect of EGFR/HER2 inhibitors based on the screening results, the expression of EGFR and HER2 proteins was examined in the cells. The level of endogenous EGFR protein did not change, but that of the phosphorylated form increased in BCAR4 fusion-overexpressing cells and xenografted tumors (Figures 2A,B). In contrast, HER2 protein expression was decreased following CD63-BCAR4 fusion. The levels of EGFR and HER2 proteins were restored by siRNA targeting the BCAR4 fusion (Figure 2C). Knockdown of the fusion gene by siRNA of CD63-BCAR4 reduced phosphorylated EGFR protein, suggesting the potential inhibition of fusion by EGFR TKI.

### 3.3 Lapatinib and canertinib inhibit BCAR4 fusion-induced migration and epithelial–mesenchymal transition signaling

**FIGURE 3:**
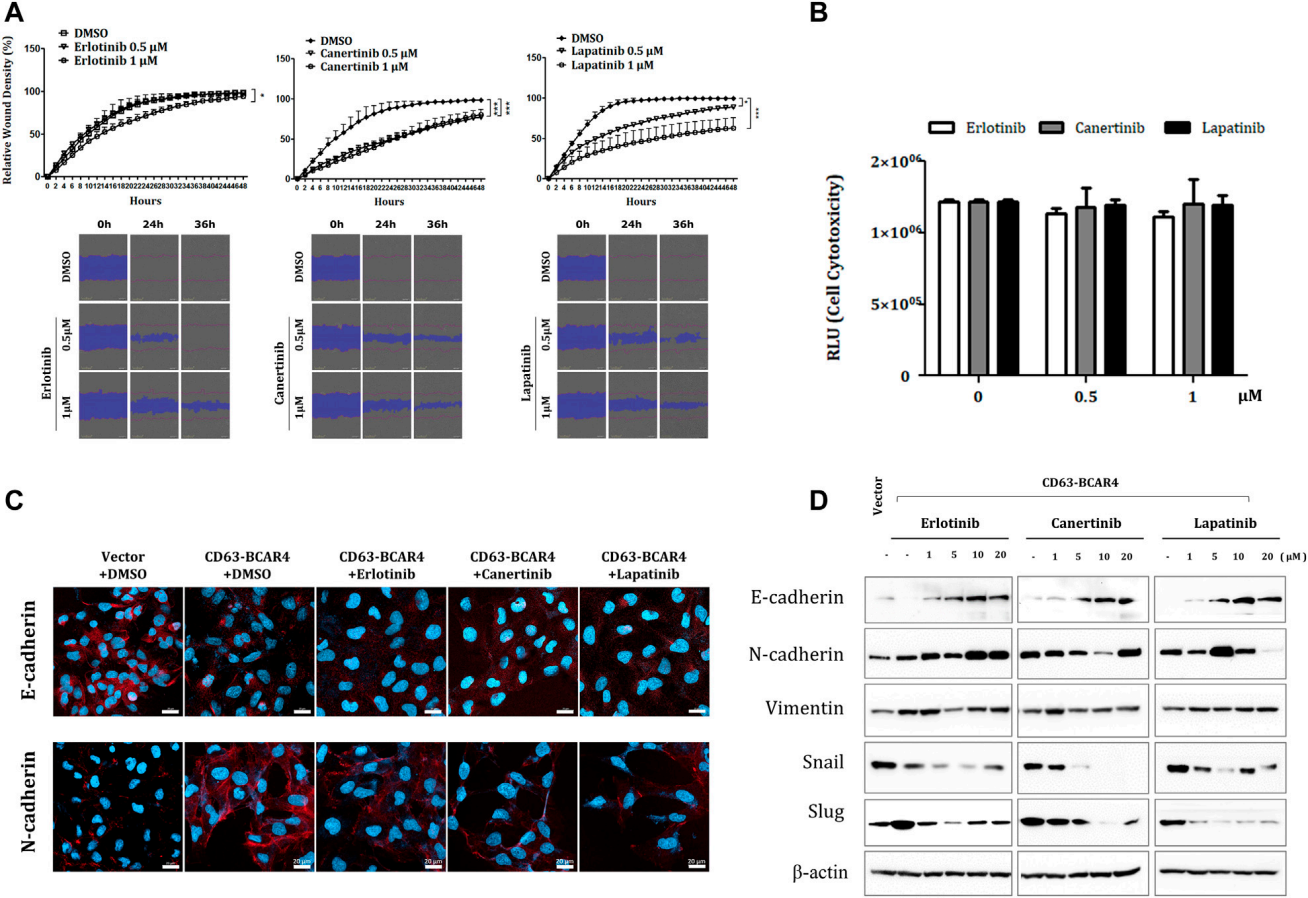
Effects of indicated drugs on cell migration and EMT signaling. **(A)** A wound healing assay was used to compare the inhibitory effects of erlotinib, lapatinib, and canertinib on cell migration of CD63-BCAR4 overexpressing BEAS-2B cells. The representative data and images from the replicated experiments are presented and statistical significances are marked with * (*p* < 0.05) and *** (*p* < 0.0005). **(B)** Cytotoxicity of each drug was assessed to confirm that motility inhibition was not due to cell death; RLU, relative light unit **(C)** Immunocytochemistry of E-cadherin and N-cadherin before and after drug treatment. **(D)** Protein levels of EMT-related molecules were evaluated using western blot analysis.

Since lapatinib is a dual inhibitor of EGFR and HER2 tyrosine kinases, we evaluated the dual inhibition of two receptor tyrosine kinases, including canertinib, a similar TKI. Compared with erlotinib, a specific inhibitor of EGFR, lapatinib and canertinib showed more potent inhibitory effects on the migration of BCAR4 fusion-overexpressing cells (Figure 3A). Since cell death was not induced at low drug concentrations, reducing cell migration can be considered a drug effect rather than a cytotoxic effect (Figure 3B). Canertinib and lapatinib treatment also decreased the expression of EMT markers, such as Slug and N-cadherin, which were induced by CD63-BCAR4. Induction of Slug by CD63-BCAR4 fusion was assessed by immunocytochemistry and western blot analysis (Supplementary Figure S1). N-cadherin expression, which was induced by CD63-BCAR4 overexpression, was decreased by EGFR/HER2 inhibitors (Figure 3C). Reduced expression of Slug and Snail and recovery of E-cadherin protein levels were observed after canertinib and lapatinib treatment (Figure 3D).

### 3.4 Canertinib targets CD63-BCAR4 overexpressing cells

**FIGURE 4:**
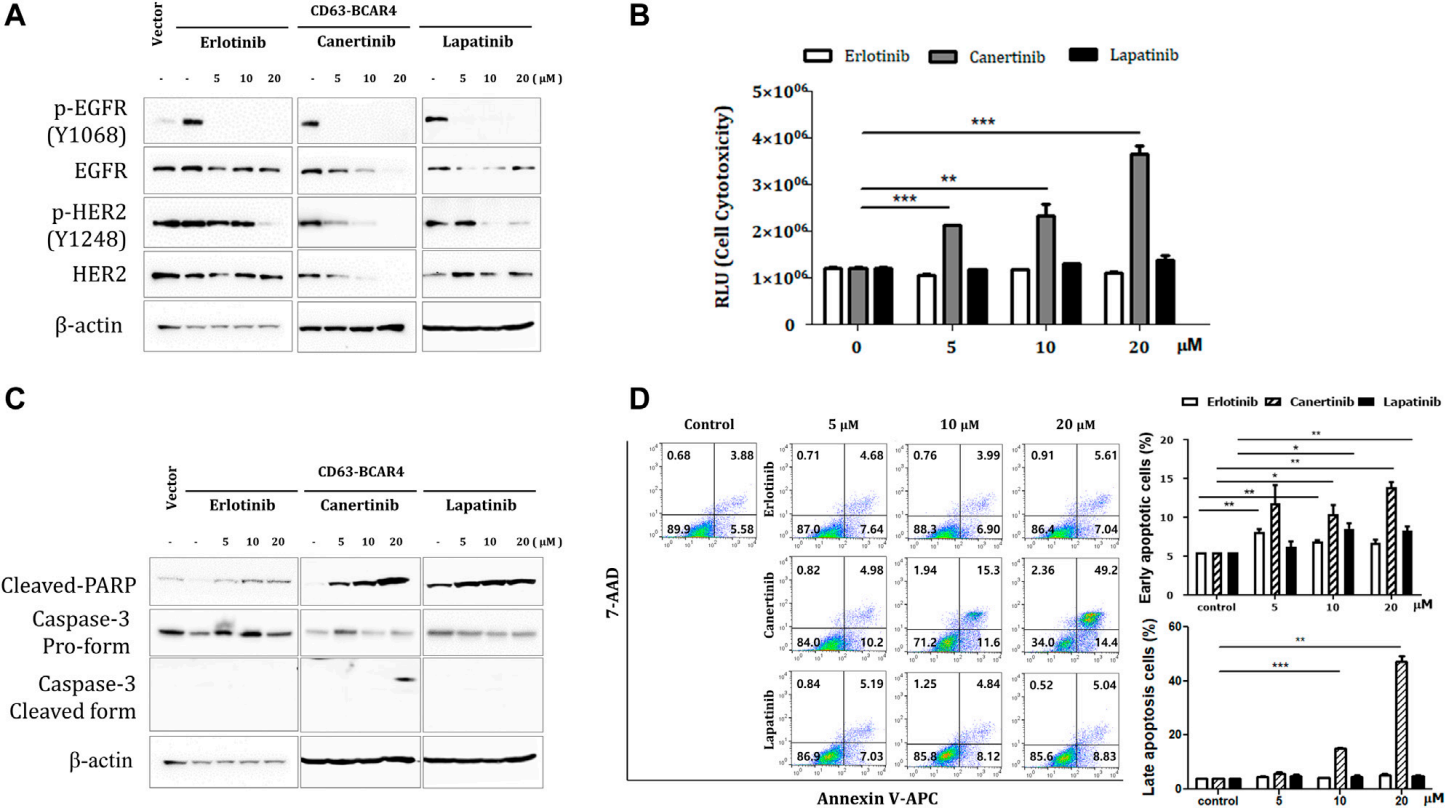
Inhibitory effects of TKIs on CD63-BCAR4. **(A)** Protein levels of EGFR, HER2, and their phosphorylated forms were examined after the indicated drug treatments. **(B)** The cytotoxicity of each drug was assessed up to a high concentration. Data represents mean ± SD of luminescence values (RLU). Statistical significances are marked with ** (*p* < 0.005) and *** (*p* < 0.0005). RLU, relative light unit. **(C)** Cleaved-PARP and caspase-3 protein were examined using western blot analysis. **(D)** Apoptosis was assessed using annexin V and 7-AAD staining. The representative images of flow cytometry profiles are shown. Early apoptosis was examined using annexin V^+^/7-AAD^-^ cells. Late apoptotic and dead cells were annexin V^+^/7-AAD^+^. Replicated experiments were performed and statistical significance are marked with * (*p* < 0.05), ** (*p* < 0.005), and *** (*p* < 0.0005).

The levels of phosphorylated EGFR decreased after treatment with erlotinib, lapatinib, and canertinib. Lapatinib and canertinib dual tyrosine kinase inhibitors of EGFR/HER2, also inhibited HER2 phosphorylation (Figure 4A). Phosphorylation of AKT and c-JUN protein was also reduced after inhibitor treatment (Supplementary Figure S2). Canertinib showed the most potent cytotoxic effect in CD63-BCAR4 overexpressing cells in a dose-dependent manner compared with that observed in response to erlotinib (Figure 4B). Apoptosis induced by canertinib and lapatinib was confirmed *via* PARP cleavage and active caspase-3 protein analysis, and annexin V staining (Figures 4C,D). The Annexin V assay revealed that early apoptosis was induced by both canertinib and lapatinib, and apoptosis was significantly induced by canertinib (early apoptosis at 20 µM, *p* = 0.0029 and 0.0068, respectively). Among the EGFR family TKIs, canertinib showed the best inhibitory effect on the migration and survival of BCAR4 fusion-overexpressing cells.

**FIGURE 5:**
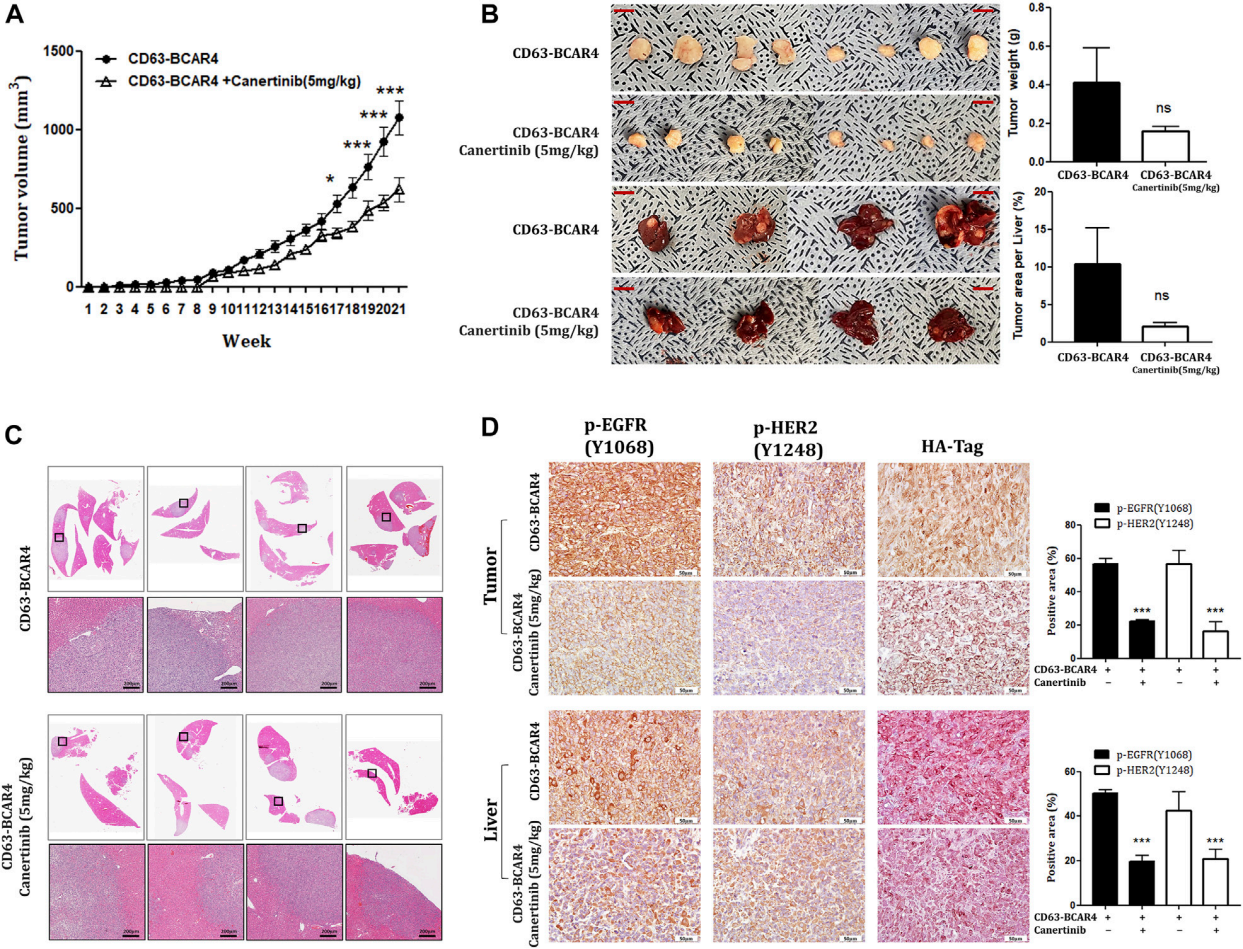
Effects of canertinib on tumor growth and metastasis in the mouse xenograft model. **(A)** Tumor growth was assessed over 20 weeks in NOG mice transplanted with CD63-BCAR4 overexpressing BEAS-2B cells with or without canertinib treatment. Tumor volumes are shown as means ± SEM (*n* = 8 in canertinib treated group, *n* = 12 in untreated group). Statistical significances are marked with * (*p* < 0.05), and *** (*p* < 0.0005). **(B)** Resected tumor specimens and livers from the subcutaneously transplanted mice. Tumor weight and metastatic tumor area per liver were compared. ns, not significant. **(C)** H&E-stained tissues from metastatic tumors in the livers of xenograft mice. **(D)** Immunohistochemistry of phosphorylated EGFR [p-EGFR (Y1068)], phosphorylated HER2 [p-HER2 (Y1248)], and HA tag proteins in tumor and liver sections from xenografted mice. The expression levels of phosphorylated proteins were compared by semi-quantitative analysis of IHC signals. Statistical significances are marked with *** (*p* < 0.0005).

We examined the effect of canertinib *in vivo* using a mouse xenograft model. Oral administration of canertinib significantly reduced tumor growth in xenograft mice injected with CD63-BCAR4-overexpressing cells (Figure 5A). Liver metastasis caused by the CD63-BCAR4 fusion gene was also reduced in canertinib-treated mice, although the difference did not reach statistical significance (Figure 5B). In addition, the area of the metastatic region in xenograft mice, as indicated by H&E staining, tended to decrease following canertinib treatment (Figure 5C). Inhibited kinase activity by canertinib was detected using immunohistochemistry of phosphorylated EGFR and HER2 proteins (Figure 5D).

### 3.5 CD63-BCAR4 is sensitive to the dual inhibition of EGFR/HER2

**FIGURE 6:**
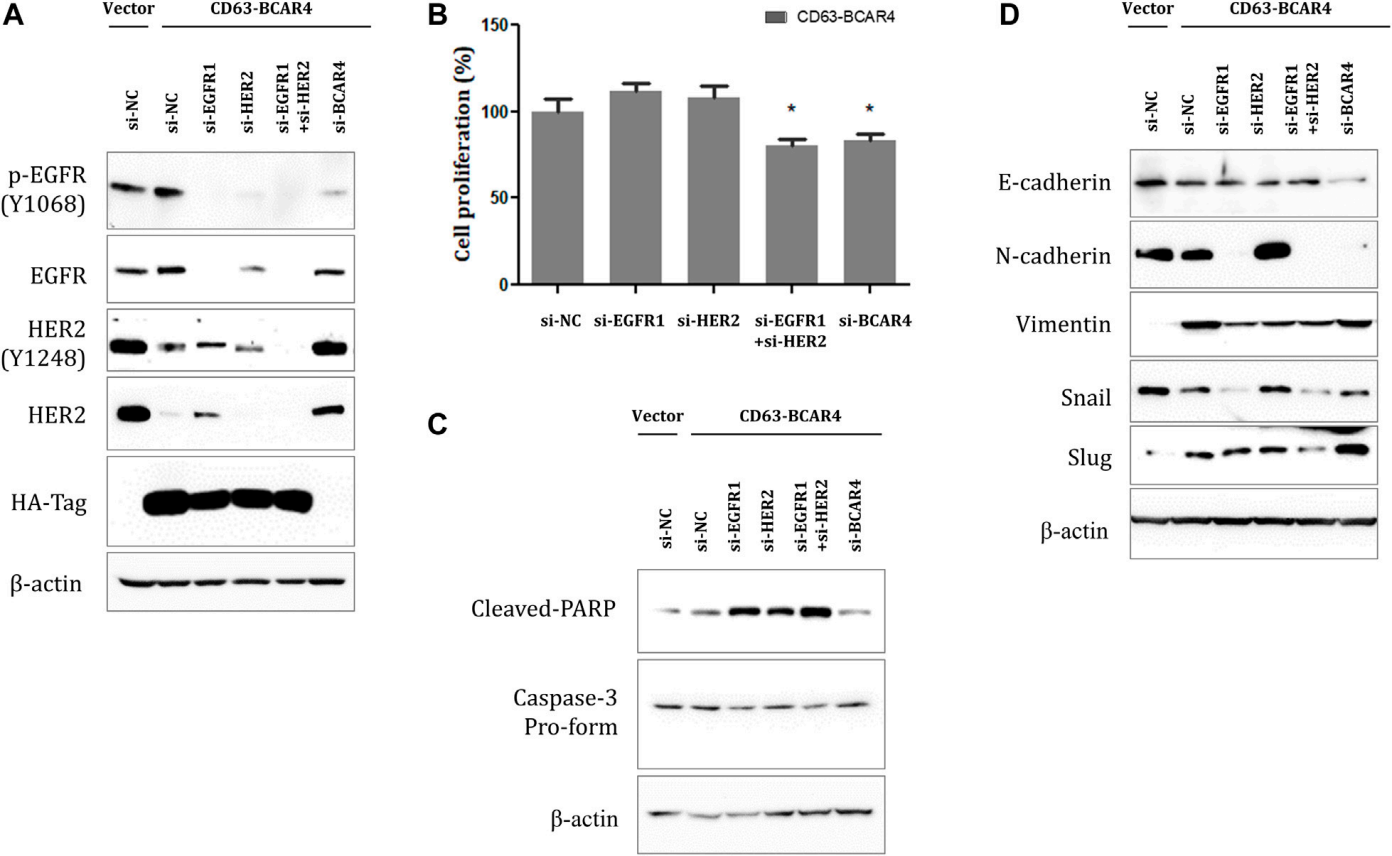
Effects of dual inhibition of EGFR/HER2 on CD63-BCAR4. **(A)** Protein levels of EGFR, HER2, and their phosphorylated forms were examined after siRNA-mediated knockdown of their expression in CD63-BCAR4 overexpressing cells. **(B)** Cell proliferation was evaluated in fusion overexpressing cells after transfection of specific siRNAs. The bar graph shows cell proliferation (%) after transfection of siRNAs compared with a negative control (si-NC). * indicates *p*-values less than 0.05. **(C)** Cleaved-PARP and caspase-3 protein levels were assessed using western blot analysis. **(D)** Effects of siRNAs targeting EGFR and/or HER2 on EMT-related proteins were evaluated using western blot analysis.

The inhibitory effect of EGFR/HER2 TKIs on CD63-BCAR4 fusion was validated using specific siRNAs targeting EGFR and HER2, respectively. Knockdown of EGFR or HER2 by siRNA was confirmed using western blot analysis (Figure 6A). Double knockdown of EGFR and HER2 by siRNAs further inhibited the proliferation of CD63-BCAR4 overexpressing cells compared with that of single knockdown cells (Figure 6B). Using relevant markers of PARP, Caspase-3, N- and E-cadherins, the enhanced effects of dual inhibition of EGFR and HER2 were observed on apoptosis and the reversal of EMT (Figures 6C,D).

## 4 Discussion

We have previously reported that CD63-BCAR4 is a novel oncogenic fusion protein in lung adenocarcinoma. Patients who harbored BCAR4 fusion commonly did not have other known activating mutations in *EGFR* and *KRAS* genes. Therefore, these patients could not receive clinical benefits from EGFR inhibitors or targeted therapy. Although TKIs targeting EGFR have shown tremendous clinical benefits in lung cancer, this is only true in patients with target activation. We have already shown enhanced metastatic activity due to CD63-BCAR4 fusion through *in vitro* and *in vivo* experiments. In the present study, we propose EGFR/HER2 TKIs as potential inhibitors of CD63-BCAR4 fusion, by demonstrating their inhibitory effects on cell migration and metastasis.

Ectopic expression of CD63-BCAR4 increased phosphorylated EGFR levels, suggesting the activation of EGFR signaling. BCAR4 has been investigated in various cancers, and its oncogenic role has been reported in breast cancer, colorectal cancer, and cervical cancer (Xing et al., 2014; Xing et al., 2015; Ouyang et al., 2017; Zou et al., 2018). Wei et al. (2019) demonstrated BCAR4 enhancement of the cell cycle and proliferation *via* activation of the EGFR signaling pathway. Upregulated EGFR and its interaction with BCAR4 were proved in glioma cells in their study. Although the mechanisms by which BCAR4 can activate EGFR have not been fully elucidated, the contribution of BCAR4 to cancer progression makes this plausible. Our results support this hypothesis by showing that BCAR4 fusion knockdown could diminish EGFR and its phosphorylation.

BCAR4, as the gene name suggests, was identified as an antiestrogen resistance gene in breast cancer. A potential relationship between BCAR4 and HER2/HER3 signaling in the development of antiestrogen resistance was reported by Godinho et al. (Godinho et al., 2010; Godinho et al., 2011). Additionally, high expression of BCAR4 sensitized breast cancer cells to the combination of lapatinib and tamoxifen (Godinho et al., 2012). EGFR and HER2 are members of the EGFR family that form homo- or heterodimers for signal transduction. Since the overexpressed CD63-BCAR4 fusion upregulated phosphorylated EGFR, it was not surprising that lapatinib and erlotinib were selected after drug library screening. Lapatinib and canertinib sufficiently inhibited EGFR and HER2 in CD63-BCAR4-overexpressing cells. The results ultimately revealed that canertinib has superior inhibitory effects on cell migration and EMT compared with those of erlotinib.

CD63-BCAR4 fusion enhanced cell migration *in vitro* and distant metastasis in an *in vivo* mouse model. EGFR inhibitors could reverse EMT stimulated by BCAR4 fusion, as shown by upregulated E-cadherin and downregulated N-cadherin and vimentin. EMT inhibition was regulated by Snail and Slug, which were inhibited by canertinib or lapatinib. Canertinib treatment reduced liver metastasis, a phenotypic hallmark of CD63-BCAR4 fusion in a mouse xenograft model. Reversing EMT by canertinib may further contribute to sensitivity to anti-cancer effects, in addition to EGFR/HER2 inhibition. Zhan et al. showed that only EGFR/HER2 heterodimers increased the invasive potential of mammary epithelial cells, which was not observed with homodimers (Zhan et al., 2006). EGFR activation in human carcinoma cell lines increases matrix metalloproteinase-9 (MMP-9) activity, which increases cell invasion by facilitating the disintegration of ECM barriers for tumor invasion (Zuo et al., 2011). We have previously shown that matrix metalloproteinase-1 (MMP-1) protein is induced by CD63-BCAR4 fusion in *in vitro* assays and *in vivo* xenograft mouse models (Bae et al., 2021). Although the interaction between BCAR4 and EGFR signaling requires further investigation, our results showed successful inhibition of canertinib and lapatinib to activate EGFR in CD63-BCAR4 overexpressed cells. The effect of BCAR4 fusion on phosphorylation and activation of EGFR needs to be determined in additional studies. Our findings suggest that dual inhibition of EGFR/HER2 may be a potential treatment option for lung cancer patients harboring oncogenic BCAR4 fusion, even in the absence of EGFR mutations.

## Data Availability

The raw data generated in this study are included in the article and Supplementary Materials, and further information can be obtained from the corresponding author upon reasonable request.
