# Supplementary material for: Oncogenic fusion of BCAR4 activates EGFR signaling and is sensitive to dual inhibition of EGFR/HER2
Source: Front Mol Biosci. 2022 Aug 23;9:952651. doi: 10.3389/fmolb.2022.952651 (PMC9445485; doi:10.3389/fmolb.2022.952651)
Supplement: Supplementary file 1 [file Image1.pdf]

Supplementary Material

1 Supplementary Methods

For immunocytochemistry, cultured cells were seeded at a density of 20,000 cells on Lab-Tek® glass chamber slides (Thermo Fisher Scientific, Rochester, NY, USA). Subsequently, the cells were fixed in a 4% formaldehyde solution for 10 min at room temperature and permeabilized by treatment with PBS containing 0.1% Triton X-100 for 5 min. The cells were blocked with 1% bovine serum albumin (BSA) for 1 h and incubated with primary antibodies overnight. The primary antibody for MMP1 (sc-137044) was purchased from Sigma-Aldrich St. Louis, MO). Antibodies for Snail (#3895) and Slug (#9585) were purchased from Cell Signaling Technology (Danvers, MA, USA). The cells were stained with a goat anti-mouse and or anti-rabbit IgG conjugated to an Alexa Fluor Plus 647 secondary antibody (Invitrogen, Waltham, MA, USA) and viewed using a confocal laser scanning microscope (LSM 900 with Airyscan2; Carl Zeiss AG, Oberkochen, Germany). Images were captured using an image analysis software (ZEN; Carl Zeiss, Munich, Germany).

For western blot analysis, cells were lysed in RIPA buffer (Cell Signaling Technology) containing protease and phosphatase inhibitor cocktails. The protein concentrations were determined using a protein assay reagent (Bio-Rad Laboratories, Hercules, CA, USA). Proteins were separated on sodium dodecyl sulfate-polyacrylamide gels using electrophoresis. The separated protein was transferred to PVDF membranes (Merck Millipore, Carrigtwohill, Ireland), and blots were blocked for 1 h with 5% skim milk (BD Biosciences, Franklin Lakes, NJ, USA) in Tris-buffered saline containing 0.1% tween-20, after which lots were incubated overnight with primary antibodies. The primary antibodies for Vimentin (#5741), phosphorylated AKT (p-AKT, #9271), c-MYC (#13987), c-JUN (#9165), phosphorylated c-JUN (#3270) were purchased from Cell Signaling Technology. Antibodies for AKT (sc-5298) and β-actin (A1978) were purchased from Santa Cruz Biotechnology (Dallas, TX, USA) and Sigma-Aldrich, respectively. Proteins were visualized using a horseradish peroxidase-conjugated secondary antibody (Cell signaling Technology) and an enhanced chemiluminescence (ECL) reagent (Bio-Rad Labaratories). β-Actin was used as a loading control.

2 Supplementary Figures

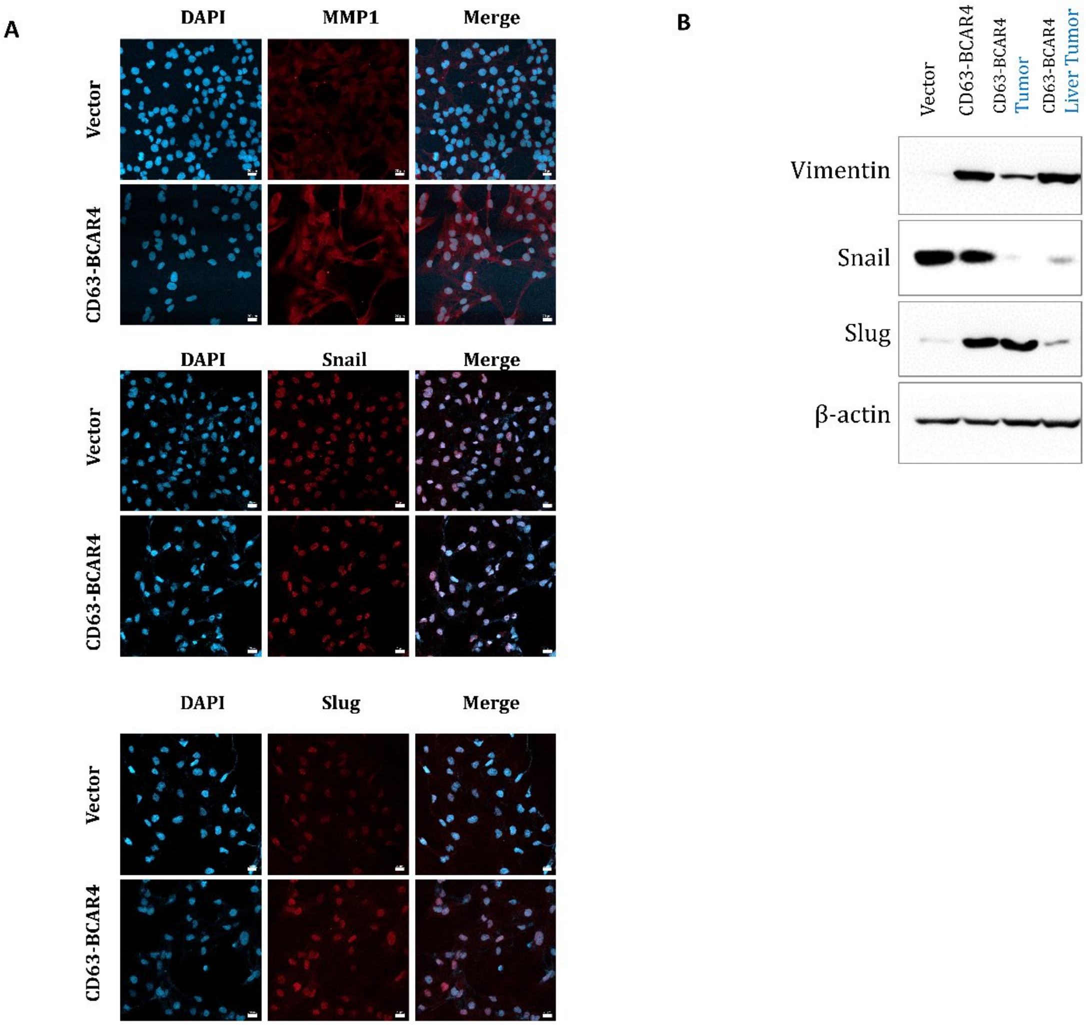

Supplementary Figure S1. Expression of EMT-related proteins. (A) Immunocytochemistry analysis revealed increased MMP1, Snail, and Slug expression in CD63-BCAR4 overexpressing cells compared with that in empty-vector expressed cells. (B) Increased Vimentin and Slug proteins in BCAR4 fusion- overexpressing cells were demonstrated by western blot analysis.

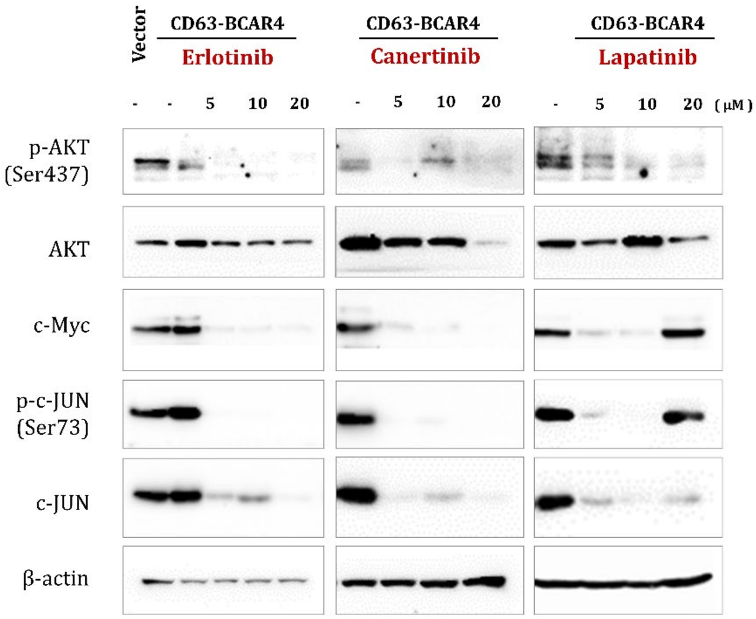

Supplementary Figure S2. Effect of inhibitors on indicated protein levels. Western blot analysis demonstrated that AKT, c-MYC, and c-JUN protein levels decreased after treatment with erlotinib, canertinib, and lapatinib.
